# Traumatic spinal cord lesions: impact of comprehensive nursing care

**Published:** 2012-11

**Authors:** Farah Roshanpour, Reza Pourmirza, Reza Khodarahmi, Alireza Saleki

**Affiliations:** ^*a*^Vice-Chancellery for Research & Technology, Kermanshah University of Medical Sciences, Kermanshah, Iran.

## Abstract

**Background:**

In the United States, about 12,000 spinal cord injuries (SCIs) are reported each year. The mean age of involved individuals is 39.5 years and 80 percent of victims are men. Most of spinal cord injuries are accompanied with brain traumatic lesions. In this way, nursing care may be important in preventing of undesired injuries.

**Methods:**

In this paper, relevant literature published in various periodicals as well as book resources are reviewed.

**Results:**

The main goal of SCI treatment is protection of the nervous system functionality using medical (e.g. corticosteroid therapy) well as surgical procedures. Hemodynamic abnormalities are common in injuries above T6, so that in the first week after injury, the patient's systolic pressure should be controlled around 85-90 mmHg. Moreover, control of body temperature is essential in these patients. Lifting of the bed and leg hanging over it are forbidden. To prevent deep vein thrombosis, low-dose heparin and elastic socks are usually used. When the patient is mechanically ventilated, administration/consumption of succinylcholine is also prohibited. Because of the high incidence of pressure-induced ulcers in the skin of these patients, continued use of mattresses (equipped with air/water flow) is recommended. Finally, It is better that the patients have no constipation.

**Conclusions:**

Patients with spinal cord injuries require complex and careful rehabilitation just after their admission. Thus, the quality of comprehensive nursing care should be evaluated, by health care systems.

**Keywords:**

Spinal Cord, Nursing, Patient


					Volume 4, Suppl. 1 Nov 2012
Publisher: Kermanshah University of Medical Sciences
URL: http://www.jivresearch.org
Copyright:
Congress of Iranian Neurosurgeons, 14-16 November 2012, Kermanshah, Iran
Paper No. 49
Traumatic spinal cord lesions: impact of comprehensive
nursing care
Farah Roshanpour a ,*
, Reza Pourmirza a
, Reza Khodarahmi a
, Alireza Saleki a
a
Vice-Chancellery for Research & Technology, Kermanshah University of Medical Sciences, Kermanshah, Iran.
Abstract:
Background: In the United States, about 12,000 spinal cord injuries (SCIs) are reported each year. The mean age of
involved individuals is 39.5 years and 80 percent of victims are men. Most of spinal cord injuries are accompanied
with brain traumatic lesions. In this way, nursing care may be important in preventing of undesired injuries.
Methods: In this paper, relevant literature published in various periodicals as well as book resources are reviewed.
Results: The main goal of SCI treatment is protection of the nervous system functionality using medical (e.g.
corticosteroid therapy) well as surgical procedures. Hemodynamic abnormalities are common in injuries above T6, so
that in the first week after injury, the patient's systolic pressure should be controlled around 85-90 mmHg. Moreover,
control of body temperature is essential in these patients. Lifting of the bed and leg hanging over it are forbidden.
To prevent deep vein thrombosis, low-dose heparin and elastic socks are usually used. When the patient is
mechanically ventilated, administration/consumption of succinylcholine is also prohibited. Because of the high
incidence of pressure-induced ulcers in the skin of these patients, continued use of mattresses (equipped with
air/water flow) is recommended. Finally, It is better that the patients have no constipation.
Conclusions: Patients with spinal cord injuries require complex and careful rehabilitation just after their admission.
Thus, the quality of comprehensive nursing care should be evaluated, by health care systems.
Keywords:
Spinal Cord, Nursing, Patient
* Corresponding Author at:
Farah Roshanpour: Vice Chancellery of Research & Technology Affairs, Kermanshah University of Medical Sciences, Kermanshah, Iran.
Tel: 08318376892, Email: Froshanpour7@ymail.com, (Roshanpour F.).

				

